# Molecular Dynamics Simulation on Orientation-Dependent Mechanical Behaviors of ZnO Single Crystals Under Nanoindentation

**DOI:** 10.3390/ma18163905

**Published:** 2025-08-21

**Authors:** Xiaolin Zhu, Jijun Li, Shiting Yang, Weiguang Zhang, Xiuxia Li, Hui Tang, Fengchao Lang, Lin Lin, Xiaohu Hou, Xueping Zhao, Jiayi Chen

**Affiliations:** 1 College of Science, Inner Mongolia University of Technology, Hohhot 010051, China; zhuxiaolin19880708@163.com (X.Z.); zhangwg@imut.edu.cn (W.Z.); lfc@imut.edu.cn (F.L.); linlin@imut.edu.cn (L.L.); chenjiayi_2012@126.com (J.C.); 2 School of Mechanical and Energy Engineering, Shanghai Technical Institute of Electronics & Information, Shanghai 201411, China; 3 School of Science and Technology, Inner Mongolia Open University, Hohhot 010011, China; tonytanghui@126.com; 4 School of Intelligent Manufacturing, Chengdu Technological University, Chengdu 611730, China; 5 Test Center, Inner Mongolia University of Technology, Hohhot 010051, China; houxiaohuhu@163.com (X.H.); xpzhao@imut.edu.cn (X.Z.)

**Keywords:** ZnO single crystal, orientation-dependent mechanical behavior, nanoindentation, molecular dynamics simulation

## Abstract

The present study aims to investigate the orientation-dependent mechanical behaviors of ZnO single crystals under nanoindentation by molecular dynamics simulation. The load–indentation depth curves, atomic displacement, shear strain and dislocations for the c-plane, m-plane and a-plane ZnO single crystals were analyzed in detail. The simulation results showed that the elastic deformation stage of the loading curves for the three oriented ZnO single crystals can be described well by the Herz elastic contact model. The Young modulus values for the c-plane, m-plane and a-plane ZnO were calculated to be 122.5 GPa, 158.3 GPa and 170.5 GPa, respectively. The onset of plastic deformation occurred first in a-plane ZnO, then in m-plane ZnO, and lastly in c-planeZnO. The atomic displacement vectors in the three oriented ZnO single crystals were in good agreement with the primary activated slip systems predicted by the maximum Schmid factor. For the c-plane ZnO, the activated pyramidal { $11\bar{2}2$ }< $11\bar{2}3$ > slip system led to a complex dislocation pattern surrounding the indenter. A U-shaped prismatic half-loop was formed in the [ $2\bar{11}0$ ] direction, confirming the activation of the prismatic { $10\bar{1}0$ }< $11\bar{2}0$ > slip system. For the m-plane ZnO, the activated prismatic { $10\bar{1}0$ }< $11\bar{2}0$ > slip system led to the preferential nucleation of dislocations along the $\left[\bar{11}20\right]$ and [ $\bar{2}110$ ] directions. A prismatic loop was formed and emitted along the [ $\bar{2}110$ ] direction, governed by a confined glide on { $10\bar{1}0$ } planes. For the a-plane ZnO, the activated prismatic { $10\bar{1}0$ }< $11\bar{2}0$ > slip system led to dislocations concentrated in the [ $\bar{1}\bar{1}20$ ] direction beneath the indentation pit, emitting a prismatic loop along this direction. Perfect dislocation (with a Burgers vector of 1/3 < $1\bar{2}10$ >) is the dominant dislocation in the three oriented ZnO single crystals. The findings are expected to deepen insights into the anisotropic mechanical properties of ZnO single crystals, offering guidance for the development and applications of ZnO-based devices.

## 1. Introduction

Wurtzite-structured ZnO is viewed as a promising wide-band-gap II-VI semiconductor due to its high exciton binding energy (~ 60 meV), excellent piezoelectric and pyroelectric properties, environmental friendliness, and high optical transparency (>90% to visible light) [1,2,3,4,5]. These excellent properties bring ZnO single crystals many opportunities for applications in piezoelectric devices, transducers, nanocomposite optoelectronic devices, transparent piezoelectric nanogenerators, and solar cells [6,7,8,9,10]. During service, ZnO-based devices are inevitably subjected to multi-directional external stresses, leading to orientation-dependent mechanical damage. Importantly, these anisotropic deformation responses exert different effects on device application performance and service life [11,12]. Therefore, understanding the orientation-dependent mechanical behavior and deformation mechanism of ZnO single crystals is essential for reliable applications in semiconductor devices.

Nanoindentation is a simple and effective technology to test the mechanical behaviors of various materials at the micro and nano scales [13,14]. Nowadays, considerable nanoindentation experiments have been conducted on ZnO single crystals to explore their mechanical properties, such as hardness [15,16], Young’s modulus [15,16,17], yield strength [18,19], and creep performance [20,21,22,23]. Coleman et al. [15] found that a-plane ( $11\bar{2}0$ ) ZnO is significantly softer than c-plane (0001) ZnO and behaves more plastically. Sung et al. performed nanoindentation and microcompression tests on c-plane (0001), a-plane ( $11\bar{2}0$ ) and m-plane ( $10\bar{1}0$ ) ZnO single crystals, and found that the nano-hardness, nano-modulus and yield strength were dependent on the crystal orientation [18]. Basu et al. carried out nanoindentation experiments to explore the deformation behavior of c-plane ( 0001 ) and a-plane ( $11\bar{2}0$ ) ZnO, and the results showed that their different hardening rates were attributed to the activation of dislocations on different sets of slip planes [19]. Lin et al. performed nanoindentation creep tests on c-plane (0001), a-plane ( $11\bar{2}0$ ) and m-plane ( $10\bar{1}0$ ) ZnO single crystals and revealed orientation-dependent creep mechanisms [21]. Juday et al. investigated the nanoindentation-induced plastic deformation in ZnO single crystals, and found that the strain and non-radiative defects generated by the indentation showed obvious orientation-dependent behavior [23]. Experimental studies have provided significant insights into the orientation-dependent mechanical behaviors of ZnO single crystals. However, the detailed evolution processes and atomic-scale deformation mechanisms of the differently oriented ZnO single crystals during nanoindentation are still unclear due to the limitations of experimental conditions.

Molecular dynamics (MD) simulations can make up for the shortcomings of experiments by displaying the evolution process of internal structures during nanoindentation [24,25]. The MD simulations have been applied to investigate orientation-dependent deformation behaviors during nanoindentation of various crystal materials (e.g., GaN, AlN and SiC) [26,27,28], while there have been limited MD studies on ZnO single crystals. Chen et al. carried out MD simulations on a c-plane (0001) ZnO single crystal to investigate its nanoindentation mechanical behaviors, and found that the dislocation loops nucleated and propagated along the slip direction due to the high local stress [29]. Hong et al. studied the effects of indenter loading rate and substrate temperature on the mechanical behaviors of c-plane (0001) ZnO single crystals by the MD method [30]. These studies have improved our understanding of the deformation mechanisms of c-plane (0001) ZnO single crystals, while the orientation-dependent mechanical behaviors of ZnO single crystals during nanoindentation are still unclear.

In this work, we chose c-plane (0001), m-plane ( $10\bar{1}0$ ) and a-plane ( $11\bar{2}0$ ) of ZnO single crystals as the indentation planes, and systematically studied the orientation-dependent mechanical behaviors of ZnO single crystals under nanoindentation by MD simulations. This work aims to achieve a better understanding of the deformation mechanisms of ZnO single crystals, and provides a reference for optimizing design and ensuring reliable application of ZnO-based devices.

## 2. Methods

### 2.1. Simulation Details

**Figure 1:**
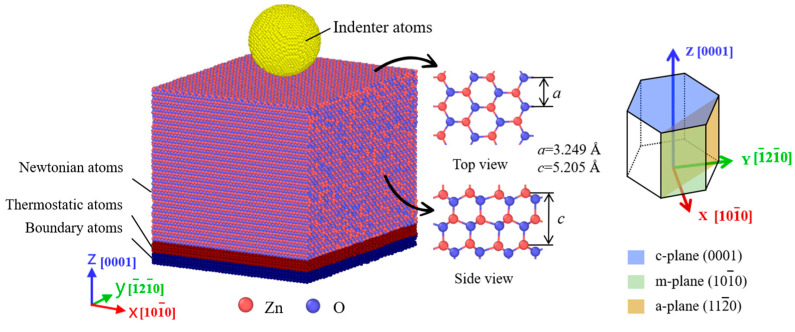
The MD model of a spherical diamond indenter against a wurtzite ZnO single crystal specimen and a schematic view of the c-plane (0001), m-plane ( $10\bar{1}0$ ) and a-planes (11 $\bar{2}0$ ) of a ZnO single crystal. The red circles represent Zn atoms, and purple circles represent O atoms. The black arrows point to the top view and side view of the crystal structure.

To explore the orientation-dependent mechanical behaviors of wurtzite ZnO single crystals, three orientations of [0001], [ $10\bar{1}0$ ] and [ $11\bar{2}0$ ] were modeled. Figure 1 shows the MD simulation model of a spherical diamond indenter against a wurtzite ZnO single crystal along the [0001] z-axis and a schematic view of the c-plane (0001), m-plane ( $10\bar{1}0$ ) and a-plane (11 $\bar{2}0$ ) of ZnO single crystals. The lattice constants are *a* = 3.249 Å and *c* = 5.205 Å for the wurtzite ZnO structure [31]. The details of the simulation parameters for the three constructed models are listed in Table 1. For simplicity, the three surfaces of ZnO single crystals are abbreviated as c-plane, m-plane and a-plane ZnO in the following.

As shown in Figure 1, the ZnO specimen was divided into three layers: the Newtonian layer, thermostatic layer, and boundary layer. To eliminate the boundary effect and reduce the size effect during nanoindentation, the four side boundaries of the specimen were set as periodic boundaries. The bottom of the specimen was set as a fixed boundary to prevent any motion of the substrate during the indentation process. Before nanoindentation, the system was equilibrated for 100 ps at 297 K in an isothermal–isobaric ensemble (NPT). Then, the equilibrated model was used for the indentation simulations. The nanoindentation process was performed in a microcanonical ensemble (NVE) with a time step of 1 fs. During the indentation process, a Langevin thermostat was employed to maintain the system temperature stably at 297 K. The indenter was initially placed 3 Å above the center of the ZnO indentation plane. During loading, the indenter moved downward along the z-axis at a speed of 30 m/s to penetrate the surface. After loading, the indenter was held at the maximum depth of 30 Å for 20 ps. During unloading, the indenter retracted back to its initial position at a speed of 60 m/s. It should be noted that, due to the inherent limitations of computational resources, the MD simulation employed shorter time scales and higher strain rates than the experiments. These settings, nevertheless, preserve the generality of the simulation results, as validated by extensive numerical and experimental studies [32,33].

### 2.2. Potential Functions

In this study, the Buckingham-type interatomic potential was employed to describe Zn-Zn, Zn-O, and O-O interactions during nanoindentation. This potential accurately reproduces the key properties of wurtzite ZnO, including lattice constants, elastic constants, surface energies, and piezoelectric coefficients. These parameters have been validated against both experimental measurements and first-principles calculations reported in prior studies [34,35].

The Buckingham-type potential function can be expressed as follows:

(1) $$U\left(r_{ij}\right)=\frac{q_{i}q_{j}}{r_{ij}}+Aexp\left(-\frac{r_{ij}}{\rho }\right)-\frac{C}{r_{ij}^{6}},$$

where U is the pair potential energy contributed by the interaction between atoms *i* and *j* with a distance of $r_{ij}$ ; $q_{i}$ and $q_{j}$ are the ionic charges; *A* and *ρ* are the parameters for the repulsive interaction; and C is the van der Waals constant. The potential parameters *A*, *ρ* and C for Zn-Zn, Zn-O, and O-O interactions are shown in Ref. [29]. Short-range interactions between atoms more than 8.5 Å apart were ignored [36]. Employing this potential, the relaxed ZnO structure exhibited optimal lattice constants of *a* = 3.258 Å, *c* = 5.222, and *c*/*a* =1.603, showing good agreement with the experimental values [31].

The diamond indenter was set as a rigid body, so the C-C interaction was ignored. The Lennard–Jones (L-J) potential was adopted to model the interactions between the diamond indenter and the ZnO model (that is, the Zn-C and O-C interactions). The L-J potential function can be written as follows:

(2) $$E=4\varepsilon \left[{\left(\frac{\sigma }{r}\right)}^{12}-{\left(\frac{\sigma }{r}\right)}^{6}\right],(r<r_{0}),$$

where *ε* is the cohesive energy, *σ* is the equilibrium distance, *r* is the distance between atoms, and the cutoff distance $r_{0}$ was set as 7 Å. The L-J potential parameters for Zn-C and O-C interactions were calculated according to the Lorentz–Berthelot mixing rules (Equations (3) and (4)) [37] and are presented in Table 2.

(3) $$\varepsilon_{ij}=\sqrt{\varepsilon_{i}\varepsilon_{j}},$$

(4) $$\sigma_{ij}=\left(\sigma_{i}+\sigma_{j}\right)/2,$$

### 2.3. Analysis Methodology

In this study, MD simulations were performed by the large-scale atomic/molecular massively parallel simulator (LAMMPS), and the results were visualized and analyzed by the Open Visualization Tool (OVITO) [38]. The dislocation extraction algorithm (DXA) was used to identify the dislocation types and their distribution patterns in the indentation-induced deformation zone.

## 3. Results and Discussion

### 3.1. Load–Indentation Depth Curves

**Figure 2:**
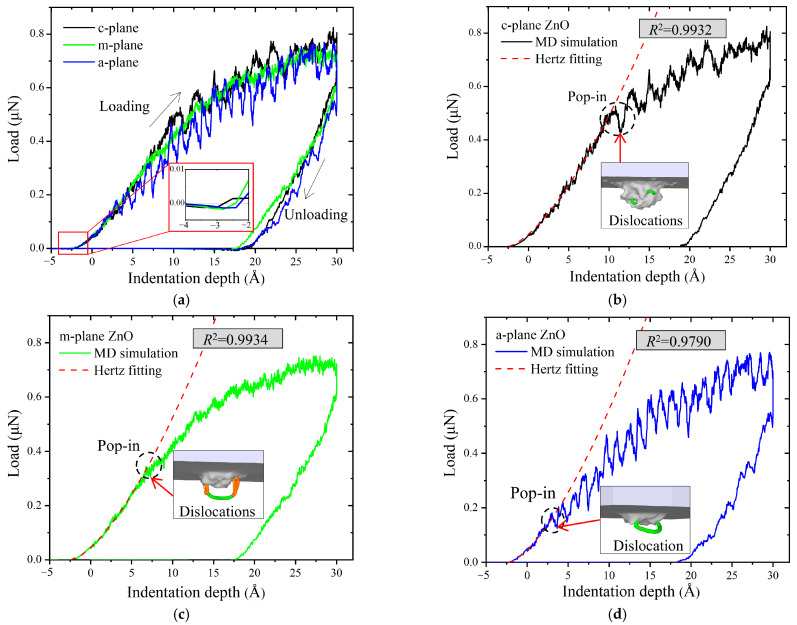
(**a**) Comparison of the load–indentation depth curves for the three oriented ZnO single crystals. The red squares represent the enlarged views of the corresponding region. Load–indentation depth curves and the corresponding Herz fitting curves (red dashed lines) on the (**b**) c-plane, (**c**) m-plane, and (**d**) a-plane ZnO single crystals under nanoindentation. The dashed circles indicate the first pop-in events. The insets represent the initial dislocations, with the red arrows pointing to the nucleated depths.

Figure 2a shows the load–indentation depth curves for the c-plane, m-plane and a-plane ZnO single crystals. With the indenter approaching the ZnO substrate, there was an attractive force between the indenter and ZnO surface initially, and then more and more atoms in the indenter were repulsed by the ZnO substrate. As shown in the insets, the loads for the c-plane, m-plane and a-plane ZnO became positive (repulsive force) from negative (attractive force) at the indentation depths of −2.6 Å, −2.4 Å and −2.3 Å, respectively. So in a strict sense, the nanoindentation was initiated at these depths for the three oriented ZnO single crystals. During loading, the load increased with the indentation depth. The loading curves for the three oriented ZnO single crystals show fluctuation features, which are attributed to the thermal motion of the atoms under nanoindentation [39]. The load–indentation depth curve for the c-plane ZnO was a little higher than those for the m-plane and a-plane ZnO, indicating that the c-plane ZnO exhibited higher hardness. During unloading, the load decreased rapidly with decreasing indentation depth. After unloading, residual indentation depths persisted in the three oriented ZnO single crystals, indicating permanent plastic deformation.

Generally, the load–indentation depth curve of elastic deformation stage can be described by the Hertz elastic contact model [28,40]:

(5) $$F=\frac{4}{3}E_{r}R^{1/2}h^{3/2},$$

where *F* is the applied load, *R* is the indenter radius, *h* is the indentation depth, and $E_{r}$ is the reduced modulus.

Figure 2b–d show the load–indentation depth curves and the corresponding Hertz elastic fitting curves for the c-plane, m-plane and a-plane ZnO single crystals, respectively. The load–indentation depth curves at the elastic stage for the three oriented ZnO single crystals could be well fitted by the Hertz elastic curves, with determination coefficients (*R*^2^) close to 1, indicating that the MD simulations in this study are reliable.

In addition, the Young’s modulus E of ZnO single crystals can be calculated as follows [41]:

(6) $$E=\left(1-\nu^{2}\right){\left[\frac{1}{E_{r}}-\frac{1-\nu_{i}^{2}}{E_{i}}\right]}^{-1},$$

where $\nu_{i}$ and $E_{i}$ are the Poisson ratio and elastic modulus of the diamond indenter. Here, $\nu_{i}$ = 0.07 and $E_{i}$ = 1141 GPa. ν is the Poisson ratio of the ZnO single crystal, and the ν values for the c-plane, m-plane and a-plane ZnO are 0.34, 0.25 and 0.25, respectively [18]. Based on Equation (6), the Young modulus of the c-plane, m-plane and a-plane ZnO were calculated to be 122.5 GPa, 158.3 GPa and 170.5 GPa, respectively. The calculated values are consistent with the experimental data, confirming that the potential employed here reliably captured the mechanical behaviors of ZnO [18,19,20]. The Young modulus of the a-plane ZnO is the largest compared to those of the c-plane and m-plane ZnO, suggesting that a-plane ZnO exhibits the greatest resistance to elastic deformation.

Generally, the onset of plastic deformation (or the first pop-in event) can be identified by the deviation of the loading curve from the Hertz elastic contact model [32,42]. As shown in Figure 2b–d, the deviation depths for the c-plane, m-plane and a-plane ZnO are 10.8 Å, 5.4 Å and 3.0 Å, and the corresponding loads are 0.527 μN, 0.266 μN and 0.18 μN, respectively. Therefore, the elastic-to-plastic deformation transition occurred first in a-plane ZnO, then in m-plane ZnO, and lastly in c-plane ZnO, which is consistent with the experimental results [15,23]. Furthermore, as shown in the insets in Figure 2b–d, the dislocations just nucleated at the corresponding deviation depths in the three oriented ZnO single crystals, indicating that their incipient plastic behaviors are induced by the dislocation nucleation.

### 3.2. Atomic Displacement and Shear Strain

**Figure 3:**
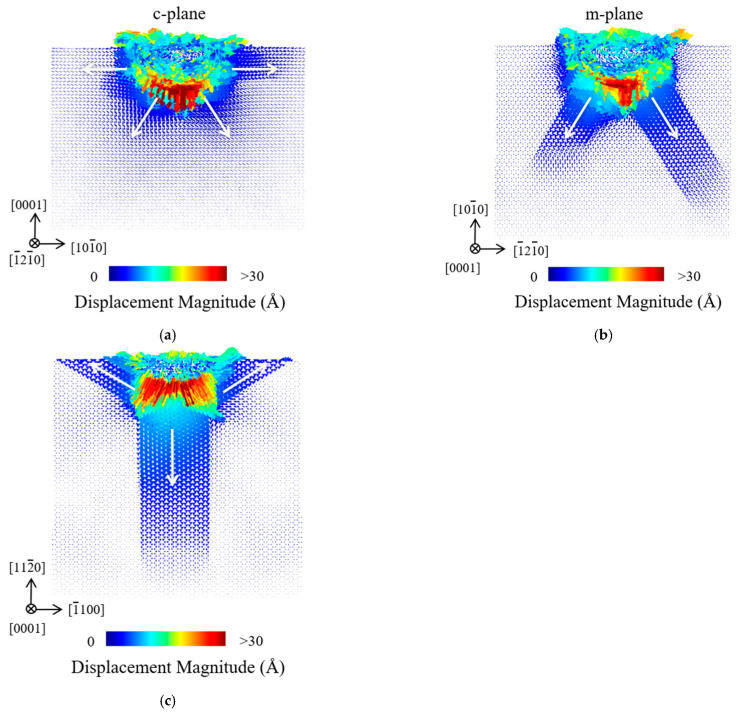
The atomic displacement vectors in the (**a**) c-plane, (**b**) m-plane and (**c**) a-plane ZnO single crystals at the maximum indentation depth of 30 Å.

Figure 3 shows the distributions of atomic displacement vectors for the c-plane, m-plane and a-plane ZnO single crystals at the maximum indentation depth of 30 Å. It can be seen that the atomic displacement is larger in the region beneath the indenter than in other deformed regions. During nanoindentation, the atoms beneath the indenter moved first and squeezed the nearby atoms, leading them to move in the directions of easier slip. Generally, the atoms near the surface of the ZnO specimen mostly moved upward to cause the pile-up, while the movement of atoms toward the substrate induced deeper subsurface damage. As shown in Figure 3a, the atoms in the c-plane ZnO moved mainly in four directions under nanoindentation, namely the [ $\bar{2}110$ ], [ $2\bar{1}\bar{1}0$ ], [ $\bar{1}\bar{1}23$ ] and [ $11\bar{2}3$ ] directions. Figure 3b shows that the atomic displacements in the m-plane ZnO were mainly along the [ $\bar{2}110$ ] and [ $\bar{1}\bar{1}20$ ] directions. As shown in Figure 3c, the atomic displacement in the a-plane ZnO was mainly along the [ $2\bar{1}\bar{1}0$ ], [ $\bar{1}2\bar{1}0$ ] and [ $\bar{1}\bar{1}20$ ] directions. This anisotropic distribution of atomic displacement vectors is due to the different slip systems activated in the three oriented ZnO single crystals [23]. Generally, the activated slip systems can be determined by the maximum Schmid factor (=cos φ cos*λ*, where φ is the angle between the slip plane normal and loading axis, and *λ* is the angle between the slip direction and loading axis) [43]. The most possible slip system for the c-plane ZnO should be { $11\bar{2}2$ }< $11\bar{2}3$ >, with a Schmid factor of 0.45 [44]. Clearly, it is consistent with the [ $\bar{1}\bar{1}23$ ] and [ $11\bar{2}3$ ] directions activated below the indentation pit in c-plane ZnO. Additionally, slip along the [ $\bar{2}110$ ] and [ $2\bar{1}\bar{1}0$ ] directions in c-plane ZnO was also activated, which is induced by the introduced shear stress components by the indenter tip curvature [45]. For the m-plane and a-plane ZnO, the most possible slip system should be { $10\bar{1}0$ } < $11\bar{2}0$ >, with a Schmid factor of 0.43 [44]. It can be seen that the primary activated slip systems predicted by the maximum Schmid factors were in good agreement with the atomic displacement vectors in our MD simulation. Furthermore, the atoms in the three oriented ZnO single crystals preferentially slipped along the < $11\bar{2}0$ > directions, because these directions are the atomic close-packed orientations for a hexagonal crystal structure.

**Figure 4:**
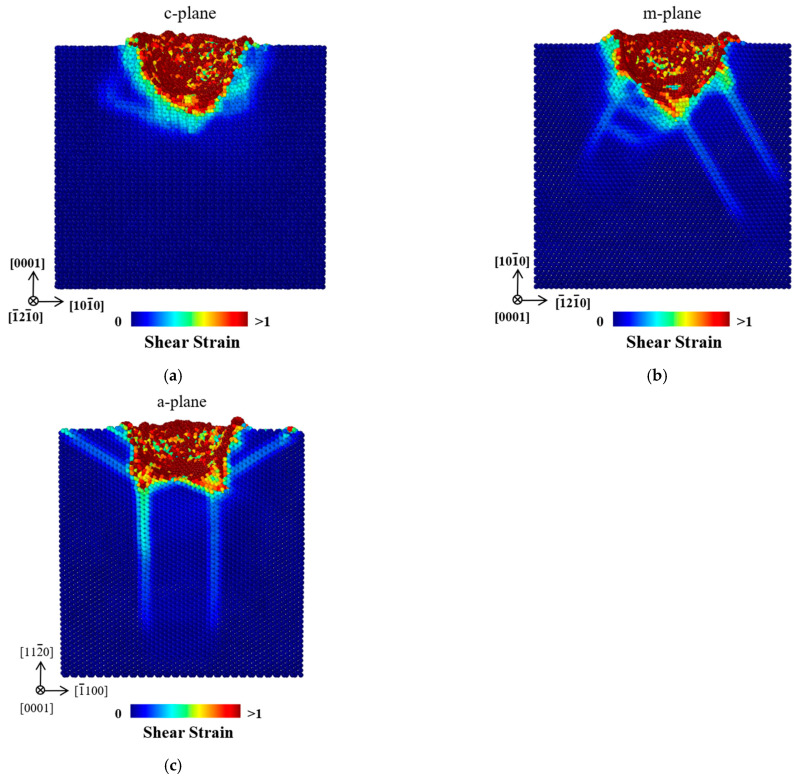
Distributions of the shear strain for the (**a**) c-plane, (**b**) m-plane, and (**c**) a-plane ZnO single crystals at the maximum indentation depth of 30 Å. The indenter can be clearly observed.

Figure 4 shows the distributions of shear strain in the c-plane, m-plane and a-plane ZnO single crystals at the maximum indentation depth of 30 Å. For the three oriented ZnO single crystals, the atoms with highest shear strain values were mainly concentrated near the indentation pit, as these regions experienced the most severe plastic deformation during nanoindentation. As shown in Figure 4a, the edge of the high shear strain zone below the indenter accorded well with the pyramidal plane, which is the activated slip plane in the c-plane ZnO. As shown in Figure 4b, the high-shear-strain bands were generated along the [ $\bar{2}110$ ] and [ $\bar{1}\bar{1}20$ ] directions in the m-plane ZnO. Figure 4c shows that the high-shear-strain bands were propagated along the [ $2\bar{1}\bar{1}0$ ], [ $\bar{1}2\bar{1}0$ ] and [ $\bar{1}\bar{1}20$ ] directions in the a-plane ZnO. The generation of high-shear-strain bands along specific directions in the m-plane and a-plane ZnO led to extensive subsurface damage. Table 3 shows the atom numbers, maximum depths and widths of high-shear-strain regions (shear strain > 0.1) for the three oriented ZnO single crystals. It can be seen that a-plane ZnO exhibited both the deepest and widest high-shear-strain region, coupled with the largest atom numbers under high shear strain, indicating that a-plane ZnO is the most susceptible to severe plastic deformation.

### 3.3. Dislocations

**Figure 5:**
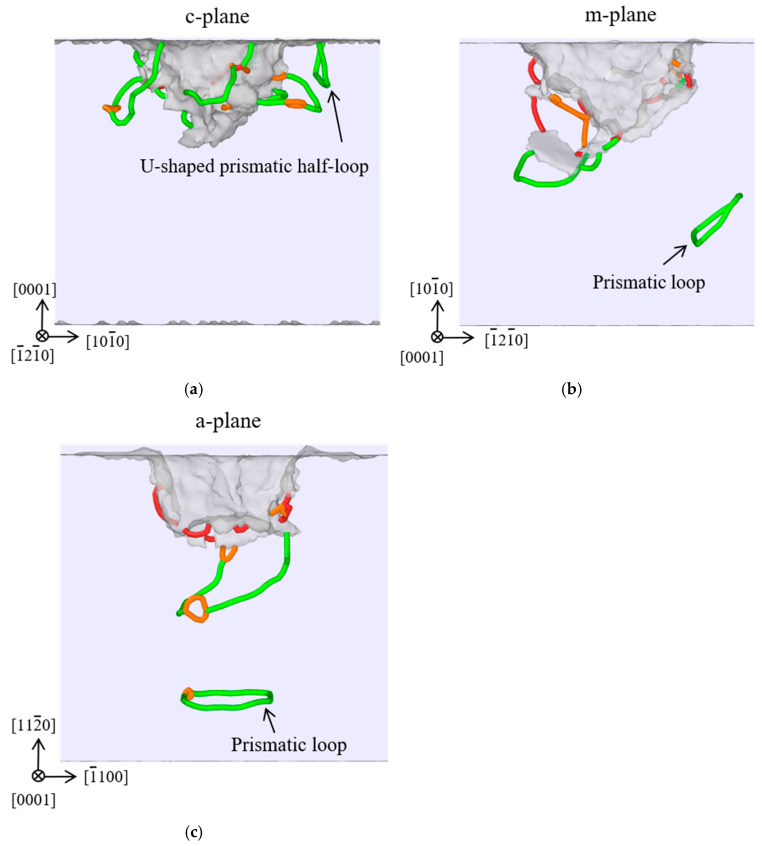
Distribution of the dislocations for the (**a**) c-plane, (**b**) m-plane, and (**c**) a-plane ZnO single crystals at the maximum indentation depth of 30 Å. The perfect dislocations are shown in green, the partial dislocations are shown in orange, and the other dislocations are shown in red.

Figure 5 shows the distributions of dislocations obtained by the DXA method for the c-plane, m-plane and a-plane ZnO single crystals at the maximum indentation depth of 30 Å. It can be seen that the identified dislocation types for the three oriented ZnO single crystals included the perfect dislocation (with a Burgers vector of 1/3< $1\bar{2}10$ >), Shockley partial dislocation (with a Burgers vector of 1/3< $1\bar{1}00$ >) and “other” dislocation. The dislocation distributions in ZnO exhibited obvious orientation dependence, owing to the different activated slip systems and formation mechanisms of dislocation. As shown in Figure 5a, when the c-plane ZnO was indented, the activated pyramidal { $11\bar{2}2$ }< $11\bar{2}3$ > slip system led to a complex dislocation pattern mainly in the < $11\bar{2}3$ > direction surrounding the indenter. A U-shaped prismatic half-loop was formed in the [ $2\bar{11}0$ ] direction, confirming the activation of the prismatic { $10\bar{1}0$ }< $11\bar{2}0$ > slip system. As shown in Figure 5b, when the m-plane ZnO was indented, the activated prismatic { $10\bar{1}0$ }< $11\bar{2}0$ > slip system led to the preferential nucleation of dislocations along the $\left[\bar{11}20\right]$ and [ $\bar{2}110$ ] directions. A prismatic loop was formed and emitted along the [ $\bar{2}110$ ] direction, governed by a confined glide on { $10\bar{1}0$ } planes. As shown in Figure 5c, when the a-plane ZnO was indented, the activated prismatic { $10\bar{1}0$ }< $11\bar{2}0$ > slip system led to the dislocations concentrated in the [ $\bar{1}\bar{1}20$ ] direction beneath the indentation pit, emitting a prismatic loop along this direction. The activated slip planes of dislocations in ZnO single crystals are consistent with the previous experimental observations [15,46], confirming the reliability of our simulation results.

**Figure 6:**
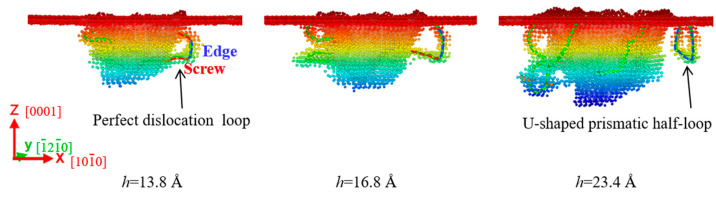
Formation of the U-shaped prismatic half-loop in the c-plane ZnO. Atoms are colored by their z-axis heights, with colors ranging from red to blue corresponding to the depth from the top surface.

Figure 6 shows the formation of the U-shaped prismatic half-loop in the c-plane ZnO during nanoindentation. At the initial stages of plastic deformation (*h* = 13.8 Å), a perfect dislocation loop consisting of two screw components (red dotted lines) and an edge component (blue dotted line) was formed on the primary { $11\bar{2}2$ } slip plane. As the indentation depth increased, the edge component slipped along the [ $11\bar{2}0$ ] direction on the initial slip plane, while the two screw components underwent cross-slip and glided laterally towards the upper surface under the resolved shear stress. When the indentation depth reached 16.8 Å and 23.4 Å, screw components climbed to the upper surface to annihilate there successively. Thus, a U-shaped half-loop with a pure edge character was formed in the prismatic { $10\bar{1}0$ } plane and slipped away from the indentation region. The lateral slip of the U-shaped prismatic half-loop was expected to lead to an atomic layer of material pile-up at the upper surface [47]. Similar U-shaped prismatic half-loops have been found in other wurtzite materials, such as GaN single crystals [26].

**Figure 7:**
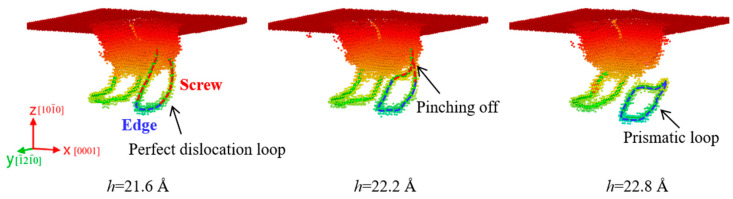
Formation of the prismatic loop in the m-plane ZnO. Atoms are colored by their z-axis heights, with colors ranging from red to blue corresponding to the depth from the top surface.

Figure 7 shows the formation of a prismatic loop in the m-plane ZnO during nanoindentation. When the indentation depth was 21.6 Å, a perfect dislocation loop consisting of two screw components and an edge component was formed in the [ $\bar{2}110$ ] direction. The screw components were perpendicular to the upper surface and had opposite inclines, while the edge component was parallel to the upper surface. As the indenter penetrated, the edge component gradually glided away along the [ $\bar{2}110$ ] direction, forming a drag force to the screw components. Accordingly, two screw components attracted each other and cross-slipped, and finally pinched off at the indentation depth of 22.2 Å. When the indentation depth increased to 22.8 Å, an independent prismatic loop was released and emitted along the [ $\bar{2}110$ ] direction into the substrate. The formation of the prismatic loop in m-plane ZnO is based on the “lasso”-like formation mechanism, which has been reported in various materials during nanoindentation [48,49]. Generally, the prismatic loop can help the substrate accommodate plastic deformation and reduce stress concentration near the indenter tip [50].

**Figure 8:**
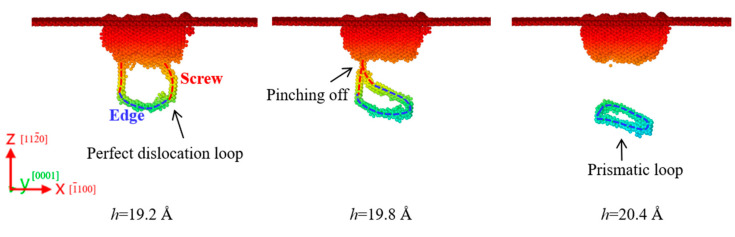
Formation of a prismatic loop in the a-plane ZnO. Atoms are colored by their z-axis heights, with colors ranging from red to blue corresponding to the depth from the top surface.

Figure 8 shows the formation of a prismatic loop in the a-plane ZnO during nanoindentation. When the indentation depth was 19.2 Å, a perfect dislocation loop with two screw components and an edge component was formed. When the indentation depth increased to 19.8 Å, the two screw components attracted each other and then pinched off. Finally, an independent prismatic loop was released at the indentation depth of 20.4 Å and emitted along the [ $\bar{1}\bar{1}20$ ] direction into the substrate. It is seen that the formation mechanism of prismatic loops in a-plane ZnO is also based onthe “lasso”-like formation mechanism. The prismatic loop in a-plane ZnO was formed earlier than that in m-plane ZnO, thereby extending the dislocation slip duration and enlarging the region of plastic deformation.

**Figure 9:**
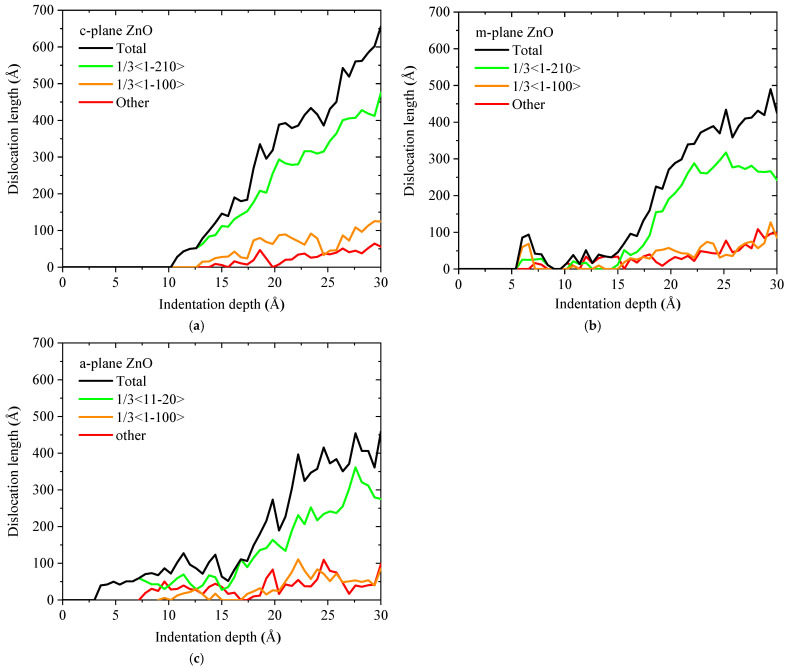
The dislocation line length-indentation depth curves in the (**a**) c-plane, (**b**) m-plane and (**c**) a-plane ZnO single crystals during the loading process.

Figure 9 shows the dislocation line length–indentation depth curves during the loading process of the c-plane, m-plane and a-plane ZnO single crystals. For the three oriented ZnO single crystals, the lengths of the perfect dislocation line, Shockley partial dislocation line, “other” dislocation line, and total dislocation line all showed increasing trends with increasing indentation depth, in spite of certain fluctuation. The total dislocation line length in the c-plane ZnO (655.7 Å) is larger than that in the m-plane (425.6 Å) and a-plane ZnO (458.7 Å) at the maximum indentation depth of 30 Å, which is due to the fact that the dislocations in c-plane ZnO had more activated slip directions. The length of the perfect dislocation line is larger than the lengths of other dislocations for the three oriented ZnO single crystals, indicating that perfect dislocation is the dominant dislocation type. This is attributed to the fact that < $1\bar{2}10$ > are the atomic close-packed directions in hexagonal lattices, requiring the lowest nanoindentation stress for slip initiation. According to the Schmid law, the critical resolved shear stress (CRSS) is proportional to the Schmid factor [51]:

(7) $$\tau_{CRSS}=\sigma \cdot cos\varphi cos\lambda$$

where σ is the indentation stress. When the slip direction is aligned with the close-packed < $1\bar{2}10$ > direction, the Schmid factor is maximized and the nanoindentation stress required to initiate slip is minimized. Accordingly, the slip of perfect dislocations along the activated slip directions dominated plastic flow, governing the orientation-dependent plastic deformation behaviors. The lengths of partial dislocations and other dislocations were confined due to suppressed dissociation under high stacking fault energy [52].

## 4. Conclusions

MD simulations of nanoindentation on c-plane, m-plane and a-plane ZnO single crystals were carried out to investigate the orientation-dependent mechanical behaviors, including the load–indentation depth curves, atomic displacement, shear strain, and dislocations. The key findings are as follows:

- The elastic deformation stages of the loading curves for the three oriented ZnO single crystals were consistent with the Herz elastic contact model. The Young moduli of c-plane, m-plane and a-plane ZnO were calculated to be 122.5 GPa, 158.3 GPa and 170.5 GPa, indicating that the a-plane ZnO has the greatest resistance to elastic deformation. The incipient plastic behaviors in the three oriented ZnO single crystals are induced by the dislocation nucleation.
- The atomic displacements in the c-plane ZnO were mainly along the [ $\bar{2}110$ ], [ $2\bar{1}\bar{1}0$ ], [ $\bar{1}\bar{1}23$ ] and [ $11\bar{2}3$ ] directions, those in the m-plane ZnO were mainly along the [ $\bar{2}110$ ] and [ $\bar{1}\bar{1}20$ ] directions, while those in the a-plane ZnO were mainly along the [ $2\bar{1}\bar{1}0$ ], [ $\bar{1}2\bar{1}0$ ] and [ $\bar{1}\bar{1}20$ ] directions. This anisotropic distribution of the atomic displacement vector is due to the different slip systems activated in the three oriented ZnO single crystals. The primary activated slip systems predicted by the maximum Schmid factors were in good agreement with the atomic displacement vectors in our MD simulation. The a-plane ZnO exhibited both the deepest and widest high-shear-strain region, coupled with the largest atom numbers under high shear strain, indicating that a-plane ZnO is the most susceptible to severe plastic deformation.
- During the loading process, a U-shaped prismatic half-loop was formed in the c-plane ZnO, and slipped away from the indenter towards the $\left[11\bar{2}0\right]$ direction. Meanwhile, a prismatic loop was formed in both the m-plane and a-plane ZnO, and emitted along the [ $2\bar{1}\bar{1}0$ ] and [ $\bar{1}\bar{1}20$ ] directions into the substrates, respectively. The formation of the prismatic loops in m-plane and a-plane ZnO are based on the “lasso”-like mechanism. The dominant dislocation type is perfect dislocation for the three oriented ZnO single crystals, which is attributed to the fact that < $1\bar{2}10$ > are the atomic close-packed directions in hexagonal lattices, requiring the lowest nanoindentation stress for slip initiation. Accordingly, the slip of perfect dislocations along the activated slip directions dominated plastic flow, governing the orientation-dependent plastic deformation behaviors.

## Figures and Tables

**Table 1:** Details of the MD simulation models and simulation parameters.

c-plane (0001) ZnO model	157.7 Å × 182.0 Å × 152.7 Å, 367,104 atoms
m- $\mathrm{plane}\;(10\bar{1}0)$ ZnO model	156.2 Å × 162.5 Å × 157.6 Å, 336,000 atoms
a- $\mathrm{plane}\;(11\bar{2}0)$ ZnO model	168.8 Å × 156.2 Å × 162.5 Å, 360,000 atoms
The radius of indenter	30 Å
Nanoindentation speed	30 m/s
Maximum nanoindentation depth	30 Å
Ambient temperature	300 K
Time step	1 fs

**Table 2:** Parameters of the L-J potential function for Zn-C and O-C interactions.

Parameter	*ε* (MeV)	*σ* (Å)
Zn-C	0.0047	3.3
O-C	0.0045	3.13

**Table 3:** Quantitative parameters of high-shear-strain regions (shear strain > 0.1) for the three oriented ZnO single crystals at the maximum indentation depth of 30 Å.

Indentation Planes	Atom Number	Max Depth (Å)	Max Width (Å)
c-plane	21,953	57.43 ± 0.5	128.84 ± 0.8
m-plane	25,314	110.61 ± 0.6	122.82 ± 0.8
a-plane	27,425	133.79 ± 0.8	157.59 ± 1.0

## Data Availability

The original contributions presented in this study are included in the article. Further inquiries can be directed to the corresponding authors.
